# Multitaxonomic Diversity Patterns along a Desert Riparian–Upland Gradient

**DOI:** 10.1371/journal.pone.0028235

**Published:** 2012-01-17

**Authors:** Candan U. Soykan, L. Arriana Brand, Leslie Ries, Juliet C. Stromberg, Christine Hass, David A. Simmons, William J. D. Patterson, John L. Sabo

**Affiliations:** 1 School of Life Sciences, Arizona State University, Tempe, Arizona, United States of America; 2 Department of Hydrology and Water Resources, University of Arizona, Sustainability of Semi-Arid Hydrology and Riparian Areas (SAHRA) Center, Tucson, Arizona, United States of America; 3 Department of Biology, University of Maryland, College Park, Maryland, United States of America; 4 Drylands Institute, Tucson, Arizona, United States of America; Duke University, United States of America

## Abstract

Riparian areas are noted for their high biodiversity, but this has rarely been tested across a wide range of taxonomic groups. We set out to describe species richness, species abundance, and community similarity patterns for 11 taxonomic groups (forbs & grasses, shrubs, trees, solpugids, spiders, scarab beetles, butterflies, lizards, birds, rodents, and mammalian carnivores) individually and for all groups combined along a riparian–upland gradient in semiarid southeastern Arizona, USA. Additionally, we assessed whether biological characteristics could explain variation in diversity along the gradient using five traits (trophic level, body size, life span, thermoregulatory mechanism, and taxonomic affiliation). At the level of individual groups diversity patterns varied along the gradient, with some having greater richness and/or abundance in riparian zones whereas others were more diverse and/or abundant in upland zones. Across all taxa combined, riparian zones contained significantly more species than the uplands. Community similarity between riparian and upland zones was low, and beta diversity was significantly greater than expected for most taxonomic groups, though biological traits explained little variance in diversity along the gradient. These results indicate heterogeneity amongst taxa in how they respond to the factors that structure ecological communities in riparian landscapes. Nevertheless, across taxonomic groups the overall pattern is one of greater species richness and abundance in riparian zones, coupled with a distinct suite of species.

## Introduction

Riparian zones are generally recognized as important for biodiversity conservation [1], [2], [3]; however, a general understanding of diversity patterns in riparian landscapes has yet to be achieved. Some studies have suggested greater species richness in riparian zones [1], others greater richness in the uplands [4], [5], [6], [7], and some showed no difference [8], [9], [10]. A recent meta-analysis detected significant heterogeneity in effect sizes—here defined as differences in species richness between riparian and adjacent uplands—across the studies it considered [11]. These contrasting results may stem from the design of most riparian–upland gradient studies that measure the species richness of one taxonomic group along a single riparian–upland gradient. Thus, it is unclear whether the differences among studies are due to taxonomy, geography, or both.

Sabo et al. [11] considered the importance of geographic location and taxonomy by contrasting richness patterns in wet versus dry climates and between studies done on animals versus plants. They found no difference in effect size—as defined above— between studies in wet and dry regions, suggesting that across species, climate alone does not drive riparian–upland gradients in species richness. Likewise, the effect sizes for animals and plants were similar, suggesting that across climate regimes taxonomy at the broad level of phyla does not drive riparian–upland gradients in species richness. However, factors besides taxonomy and climate varied among studies, complicating comparisons [11]. Few studies have documented patterns of species richness across the same riparian–upland transition for a representative suite of taxonomic groups.

A sampling program for multiple taxonomic groups along a riparian–upland transition in a single geographic region would control for site-to-site variability in environmental conditions, shedding light on the importance of taxonomic identity for structuring communities along riparian–upland gradients. Moreover, a variety of biological traits such as body size, mobility, diet, lifespan, reproductive allocation patterns, morphology, and physiology (e.g., water and temperature regulation) may help explain differential responses to changes in biotic and abiotic conditions along the riparian–upland gradient [12], [13].

Although their results for richness were equivocal, Sabo et al. [11] did detect significant turnover or beta-diversity along riparian–upland gradients. A multitaxonomic sampling program would test the generality of this result across taxonomic groups while controlling for environmental variables. Moreover, it would be possible to examine patterns in species abundance, providing a more complete description of species diversity patterns along the riparian–upland gradient.

To explore diversity patterns along a single riparian–upland gradient we synthesized data collected from eleven taxonomic groups (forbs & grasses, shrubs, trees, solpugids, spiders, scarab beetles, butterflies, lizards, birds, rodents, and mammalian carnivores) along the upper San Pedro River in southeastern Arizona, USA. These groups are phylogenetically diverse, cover multiple trophic levels, and exhibit a variety of life history characteristics. Our specific objectives were: 1) to assess how species diversity (species richness, abundance, and turnover) varied within and among taxonomic groups along a riparian–upland gradient and 2) to evaluate whether biological traits (e.g., body size) explained these diversity patterns along the gradient.

### Study System

**Figure 1:**
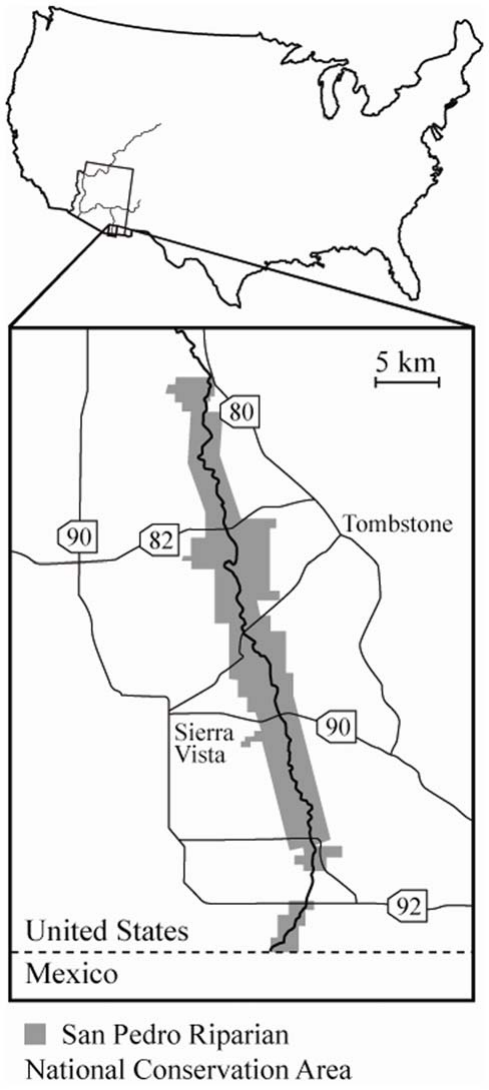
Map of the upper San Pedro River. The river is located in the Chihuahuan Desert of southeastern Arizona, USA. Note the extent of the San Pedro Riparian National Conservation Area, shaded gray.

**Figure 2:**
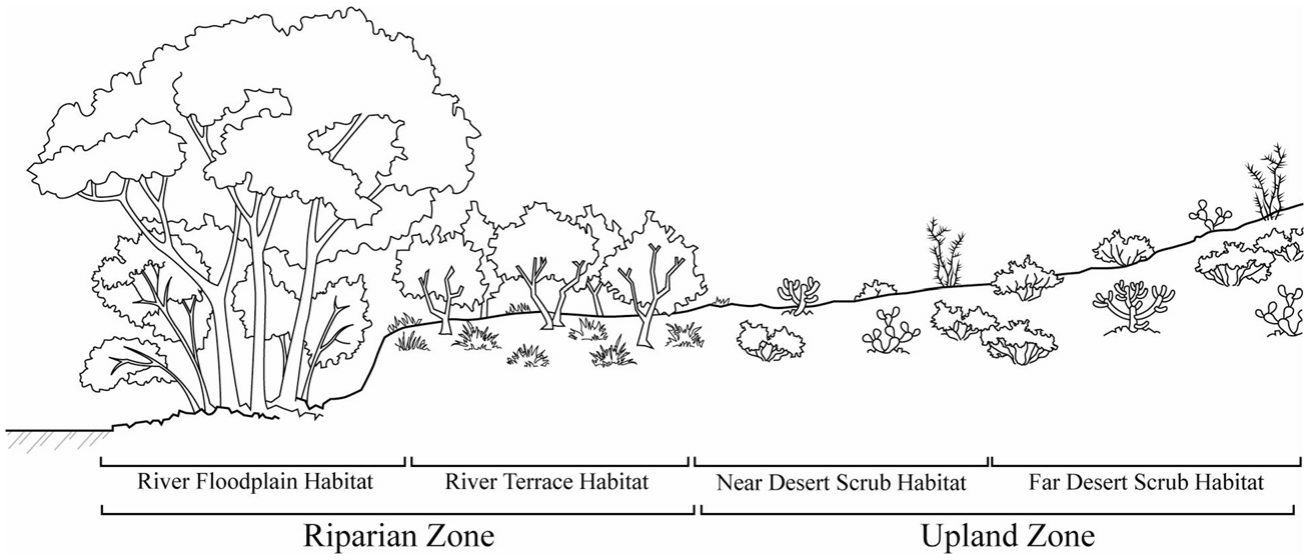
Schematic of the riparian–upland gradient. Note the four habitat types and two zones located along the upper San Pedro River.

All studies included in this synthesis were conducted in the San Pedro Riparian National Conservation Area along the upper San Pedro River in southeastern Arizona (Figure 1). Located at ∼1200 meters above sea level, the region is dominated by Chihuahuan Desert Scrub, but features high abiotic and biotic spatial heterogeneity due to the presence of the river and surrounding mountains. In the vicinity of the river, this heterogeneity includes distinctive habitat types such as cottonwood-willow forests (*Populus fremontii*, *Salix gooddingii*), mesquite bosques (*Prosopsis velutina*), sacaton grasslands (*Sporobolus wrightii*), and desert scrub (Figure 2). Although numerous factors such as flood inundation, soil type, and elevation above sea level interact to determine habitat type, water table depth is a primary control [14]. Generally, cottonwood-willow forests occurs within the river floodplain in areas where the water table is close to the ground surface, mesquite bosques and sacaton grasslands dominate river terraces with intermediate water table depth, and desert scrub occurs in upland areas where the water table is beyond the reach of plant roots.

In addition to its role as an ecological study system, the San Pedro watershed has drawn the attention of conservationists because it is one of the last unimpounded perennial rivers in the American Southwest [15], harboring speciose assemblages of mammals [16], birds [17], herpetofauna [18], invertebrates [19], and plants [20]. The primary factor threatening the river (and associated riparian zone) is groundwater decline caused by pumping of the aquifer to meet the needs of a growing human population [15]. How river drying would affect biodiversity depends, in large part, on the strength of diversity gradients and/or turnover in species pools along the riparian–upland transition.

We hypothesize that six main factors interact to structure ecological communities along the upper San Pedro River: environmental stress, disturbance, competition, predation, biological productivity, and habitat structural complexity. Environmental stress, which is inversely proportional to water availability, is lowest in the floodplain habitat type, intermediate in the river terrace, and highest in the desert scrub. Flood disturbance follows an opposite pattern, with levels dropping rapidly from floodplain to terrace habitat types, and the level in the desert scrub being effectively zero [15]. Competition and predation are highest in floodplain and decline gradually along the gradient into the uplands [15], [16], [21]. Biological productivity and habitat structural complexity are highest in the floodplain habitat type, decline in the river terrace, and then drop further in the desert scrub [15], [16].

## Methods

All necessary permits were obtained for the described field studies, including a protocol approved by the Arizona State University Institutional Animal Care and Use Committee (IACUC Protocol # 03-684R) and a research permit approved by the Arizona Game and Fish Department (permit # SP7194998), along with verbal approval from the Sierra Vista office of the Bureau of Land Management, and the Environmental and Natural Resources Division of Fort Huachuca/USAIC.

Given the range of taxa included in this study, and that the studies were initiated separately and for different purposes, a variety of survey methods were used to gather information on species diversity in the different habitat types (see Supporting Information: Detailed Methods S1). As such they vary in terms of survey effort, duration, and extent. We collected data for the trees, shrubs, and forbs/grasses in the river floodplain habitat type (FP), predominantly cottonwood-willow forests, and on river terraces (RT)—mesquite bosques and sacaton grasslands. We also collected data for the birds, butterflies, and lizards in these habitat types and from upland areas within 1 km of the river (near-river desert scrub – NS). We collected data for the solpugids, spiders, scarab beetles, and small mammals from the three habitat types described above, along with samples from upland areas greater than 1 km from the river (desert scrub far from the river – FS). We collected data for mammalian carnivores from FP, RT, and upland areas, without categorizing the upland areas as near- or far-from the river. For each group, we collected data from one or more plots/transects for each habitat type from a set of four or more sites along the river, leading to replication of our effort across habitat types and sites (Table 1). Habitat types at a site were separated by ∼10–1000 meters, while sites were separated by ∼1–10 kilometers. For the purposes of this study, we distinguish between ‘habitat types’ and ‘zones’ (Figure 2). Habitat type refers to the four categories described above (FP, RT, NS, FS), while zone combines the FP and RT into a Riparian Zone and NS and FS into an Upland Zone.

**Table 1:** Overview of survey methods, habitat types sampled, number of sites sampled, and sampling dates for the eleven taxonomic groups discussed in this paper.

Taxonomic Group	Method	Habitat Types	# of Sites	Dates (MM/YY)
Forbs & Grasses	plot surveys	FP, RT	10*	08/01–09/02
Shrubs	transects	FP, RT	10	05/01–09/02
Trees	plot surveys	FP, RT	10	05/01–09/02
Solpugids	pitfalls	FP, RT, NS, FS	4	05/04–10/05
Spiders	pitfalls	FP, RT, NS, FS	4	05/04–10/05
Scarab Beetles	pitfalls	FP, RT, NS, FS	4	05/04–10/05
Butterflies	quadrat surveys	FP, RT, NS	9	08/98–07/01
Lizards	transects & pitfalls	FP, RT, NS	4	05/02–08/04
Birds	point-count	FP, RT, UP	15	05/98–07/01
Rodents	live-trapping	FP, RT, NS, FS	7	07/03–10/05
Mammalian Carnivores	transects	FP, RT, UP	12	10/98–01/00

Note: FP = river floodplain habitat type; RT = river terrace habitat type; NS = upland areas within 1 km of the river; FS = upland areas greater than 1 km from the river; UP = upland areas.

*There were 11 sites surveyed for forbs/grasses in the RT habitat type in 2002.

We analyzed diversity patterns along the riparian–upland gradient at three different levels of organization (within taxon, across-taxa, and based on five biologically-defined trait groups).

### Taxon-Level Analyses

We compared species richness among habitat types or zones using estimates equivalent to those based on traditional resampling techniques. We rarefied samples down to the largest shared number of individuals or samples using the Mau Tau function implemented in EstimateS [22]. We used individual-based rarefaction if individual-based methods were used to sample a taxon (e.g., mammalian carnivores), whereas we used sample-based rarefaction if sample-based methods were used to sample a taxon (e.g., forbs & grasses, shrubs, trees, solpugids, spiders, scarab beetles, butterflies, lizards, birds, and rodents). We combined samples from a given habitat type across sites, which increases sample sizes, but adds heterogeneity to the analysis due to site-to-site differences in environmental conditions.

We estimated species abundance by bootstrapping sample-level abundance values for each habitat type or zone [23]. This resampling method allowed us to calculate a bootstrap mean and 95% confidence intervals for each taxonomic group in each habitat type or zone. We standardized abundances by effort within groups, but since we used multiple methods for sampling the disparate taxa included in this study the standardized abundance values are not comparable among taxonomic groups. We resampled abundances using the PopTools plugin for Microsoft Excel [24].

We used the classic Sørensen index of community similarity, calculated with EstimateS software, to assess changes in community composition among habitat types or between zones. The Sørensen index is interpreted as representing the mean proportion of shared species between two samples (i.e., the number of species common to both samples divided by the average number of species in each sample). For this analysis, we lumped together samples from the same habitat type (or zone) at different sites so that the similarity metric measured changes between habitat types (or zones) at the regional scale.

Additive partitioning of species richness, done using the program PARTITION [25], provides a complementary way of measuring changes in community composition across habitat types [26]. Additive partitioning calculates alpha or within-habitat diversity as the mean number of species per habitat type (i.e., averaging samples from a given habitat type across sites), and beta or among-habitat diversity as the mean number of species that are added to the regional total by sampling across habitat types. We used individual-based randomization to compare observed turnover with that expected based on a random distribution of individuals among samples.

### Cross-Taxa Analyses

In order to avoid the pitfalls associated with qualitative approaches to summarizing results across studies (e.g., ‘vote counting’), we conducted a meta-analysis on the results of the individual taxon-level studies [27]. A meta-analysis is a statistically rigorous method that relies on the effect size of a factor rather than its significance level. Effect sizes can be defined in a variety of inter-related ways [28], but most effect size measures contain information about the mean difference, the pooled variance, and the sample size. Additionally, effect sizes like the one we employed can be further corrected for small sample biases. The result is a statistical measure of the combined (cumulative) effect size of a group of studies that factors in the quality (i.e., low variance, high sample size) of each study. Here we employ meta-analytic methods to combine results across individual taxon-level studies, allowing for statistical inference about the community as a whole.

As input for the meta-analyses we used Mau Tau richness and bootstrapped abundance estimates. Due to data availability we focused on two comparisons, riparian versus upland zones and river floodplain versus river terrace habitat types. We calculated an unweighted effect size for each taxonomic group as follows:
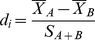
(1)where, *d_i_* is the unweighted effect size of the *i*th record, 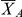 and 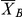 are average richness or abundance estimates in the riparian zone (river floodplain habitat type) and upland zone (river terrace habitat type), respectively, and *S_A+B_* is the pooled standard deviation of mean estimates from the two zones or habitat types for each taxonomic group. Values that were greater than zero indicated greater richness or abundance in the riparian zone or river floodplain habitat type. We then weighted the effect size estimates according to the number of samples collected for each taxonomic group. This gave taxa that were more thoroughly-sampled greater weight in the meta-analysis. We used the meta-analysis program MIX version 1.7 [29], [30] to calculate average weighted effect sizes across taxa using random effects models and Hedge's G as the association measure. We chose to run random effects models because sampling methods differed among taxa, and used Hedge's G to correct for small sample bias and because it is appropriate for experimental studies—e.g., control/treatment or two-level single factor [27].

We could not analyze similarity estimates using a meta-analytic approach because they had no measure of variance. Instead we averaged the taxon-level Sørensen similarities to calculate a mean, cross-taxa similarity and accompanying variance estimate for each habitat type pair.

### Trait-Level Analyses

We used our collective expertise to assign each of the 11 taxonomic groups to various trait categories (see Supporting Information: Table S1). The five traits that we investigated—trophic level, body size, life span, thermoregulatory mechanism, and taxonomic affiliation—each required a separate analysis. Each trait-level analysis involved a non-parametric Kruskal-Wallis ANOVA, with the treatment levels representing different categories for traits (e.g., exotherm vs. endotherm for thermoregulatory mechanism). We used the unweighted effect size estimates for each taxonomic group from the meta-analyses of richness and abundance as response variables. As with the meta-analysis, we focused on two comparisons, riparian versus upland zones and river floodplain versus river terrace habitat types.

## Results

The number of surveys/samples, the number of individuals, and the number of species detected by habitat type are presented in the Supporting Information: Table S2.

### Taxon-Level Analyses – Habitat Types

**Figure 3:**
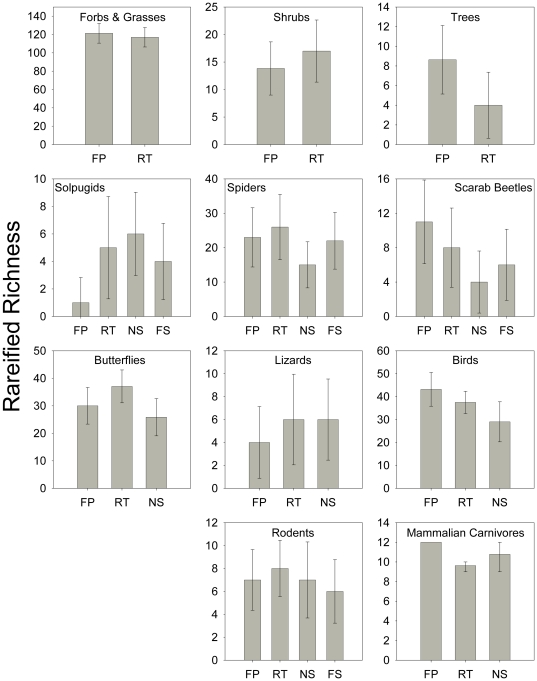
Richness by habitat type for each taxonomic group. Mau Tau rarefied richness values (±95% confidence intervals) in four different habitat types for the eleven taxa sampled along the upper San Pedro River (abbreviations as in Table 1). The only values that had non-overlapping 95% confidence intervals were solpugids in NS and FP habitat types.

When comparing individual habitat types (FP, RT, NS, FS), six taxa had a trend of higher Mau Tau rarefied richness in the river floodplain habitat type (forbs and grasses, trees, scarab beetles, birds, and mammalian carnivores), four taxa had a trend of higher rarefied richness in the river terrace habitat type (shrubs, spiders, butterflies, and rodents), one taxon, solpugids, showed a trend of higher rarefied richness in the NS habitat type, and one taxon, lizards, had equally high estimates in RT and NS habitat types (Figure 3). However, the only comparison that was statistically significant was between solpugids in NS and FP habitat types.

**Figure 4:**
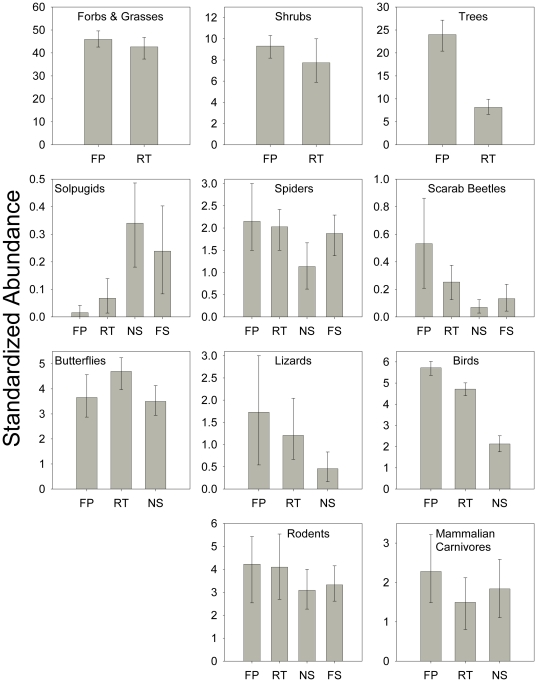
Abundance by habitat type for each taxonomic group. Bootstrapped abundance estimates (±95% confidence intervals) in four different habitat types for the eleven taxa sampled along the upper San Pedro River (abbreviations as in Table 1).

Two taxonomic groups (trees and birds) were significantly more abundant in river floodplains than any other habitat type (Figure 4. An additional seven groups (forbs and grasses, shrubs, spiders, scarab beetles, lizards, rodents, and mammalian carnivores) were most abundant in the river floodplain habitat type, but the differences were not statistically significant. Scarabs were significantly more abundant in FP and RT than NS habitat types. The difference in bird abundance between RT and NS habitat types was also statistically significant. Butterflies were most abundant in the RT habitat type, but the differences were not statistically significant. Solpugids, on the other hand, were significantly more abundant in NS and FS than in FP habitat types; the difference between NS and RT was also statistically significant.

Forty-three percent (24/56) of the similarity comparisons were below 0.5 indicating that many habitat types had less than half of their sampled species in common (Table 2). Sørensen similarity values tended to be highest for adjacent habitat types (mean = 0.61), declined for habitat types separated by an intermediate habitat type (mean = 0.50), and dropped to low levels (mean = 0.31) when distant FP and FS habitat types were compared. These differences were not statistically significant, however, due to the variability in the data.

**Table 2:** Sørensen similarity values for habitat type pairs.

Taxonomic Group	FP-RT	RT-NS	NS-FS	FP-NS	RT-FS	FP-FS	Riparian-Upland
Forbs & Grasses 2001	0.699	NA	NA	NA	NA	NA	NA
Forbs & Grasses 2002	0.609	NA	NA	NA	NA	NA	NA
Shrubs	0.765	NA	NA	NA	NA	NA	NA
Trees	0.429	NA	NA	NA	NA	NA	NA
Solpugids	0.333	0.545	0.800	0.286	0.667	0.400	0.545
Spiders	0.308	0.471	0.364	0.121	0.308	0.105	0.383
Scarab Beetles	0.316	0.000	0.400	0.000	0.143	0.118	0.166
Butterflies 1999	0.723	0.711	NA	0.750	NA	NA	0.653
Butterflies 2000	0.708	0.744	NA	0.667	NA	NA	0.708
Lizards	0.400	0.333	NA	0.400	NA	NA	0.428
Birds	0.743	0.553	NA	0.437	NA	NA	0.442
Rodents	0.933	0.800	0.923	0.714	0.714	0.615	0.800
Mammalian Carnivores	0.909	0.909	NA	1.000	NA	NA	1.000
**Mean**	0.606	0.563	0.622	0.486	0.458	0.310	0.569
**Variance**	0.050	0.076	0.079	0.105	0.077	0.060	0.062
**Upper 95% CI**	0.741	0.775	1.070	0.735	0.899	0.699	0.761
**Lower 95% CI**	0.471	0.351	0.173	0.237	0.017	−0.080	0.377

Note: NA = Not Assessed; other abbreviations as in Table 1.

The among-habitat beta diversity calculated using additive diversity partitioning was greater than expected for all taxa except mammalian carnivores (Table 3). For eight of the taxa (forbs and grasses, shrubs, trees, spiders, scarabs, birds, lizards, and rodents) the difference between observed and expected levels of beta diversity was statistically significant.

**Table 3:** Observed and expected beta diversity values based on the additive partitioning method of Veech et al. [26].

Taxononmic Group	Observed	Expected	p-value*
Forbs & Grasses	35.00	9.08	<0.001
Shrubs	4.00	2.04	<0.001
Trees	4.00	1.82	<0.001
Solpugids	4.00	3.94	0.197
Spiders	37.50	33.17	<0.001
Scarab Beetles	14.75	11.72	<0.001
Butterflies	11.00	10.25	0.063
Lizards	5.67	4.72	<0.001
Birds	29.33	19.48	<0.001
Rodents	2.00	0.67	<0.001
Mammalian Carnivores	0.67	0.88	0.869

*The probability of observing the measured values of beta diversity is based on the randomization of individuals among samples.

### Taxon-Level Analyses – Zones

**Figure 5:**
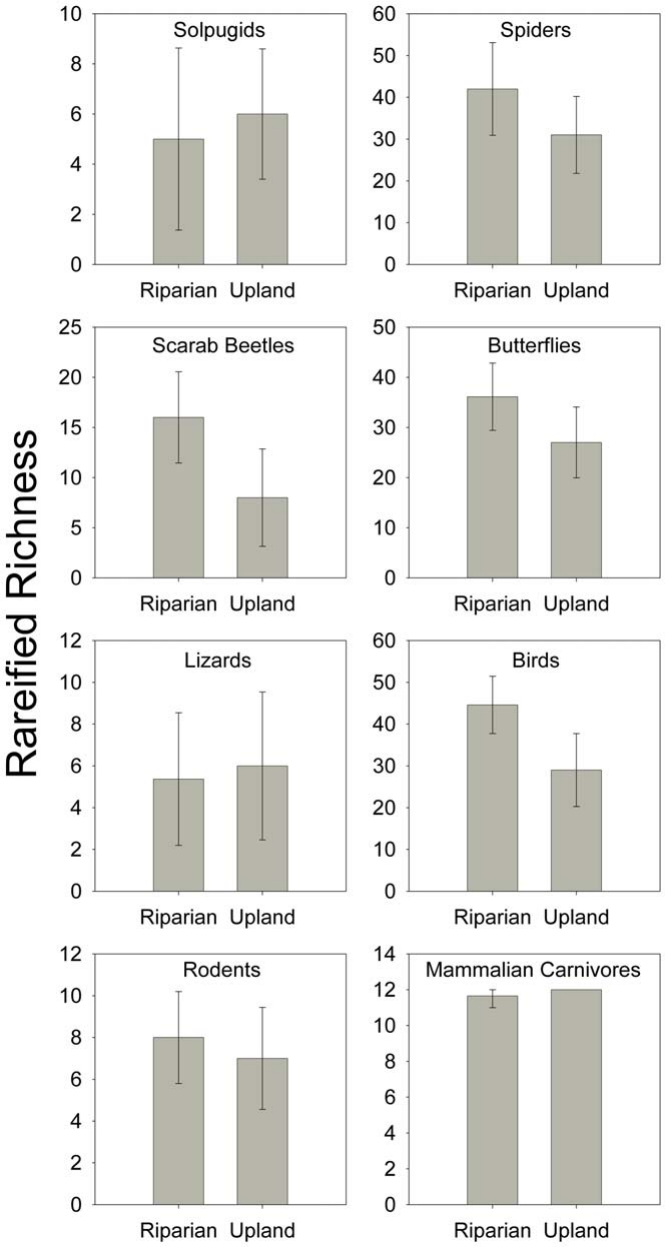
Richness by zone for each taxonomic group. Mau Tau rarefied richness values (±95% confidence intervals) for the eight taxa sampled in both riparian and upland zones along the upper San Pedro River. The only values that had non-overlapping 95% confidence intervals were birds in riparian and upland zones.

When comparing zones (riparian, upland), five of the eight taxonomic groups (spiders, beetles, butterflies, birds, and rodents) had a trend of higher rarefied richness in riparian zones, and three groups (solpugids, lizards, mammalian carnivores) had a trend of higher rarefied richness in upland zones (Figure 5). However, the only comparison that approached statistical significance (p≈0.05) was for the birds.

**Figure 6:**
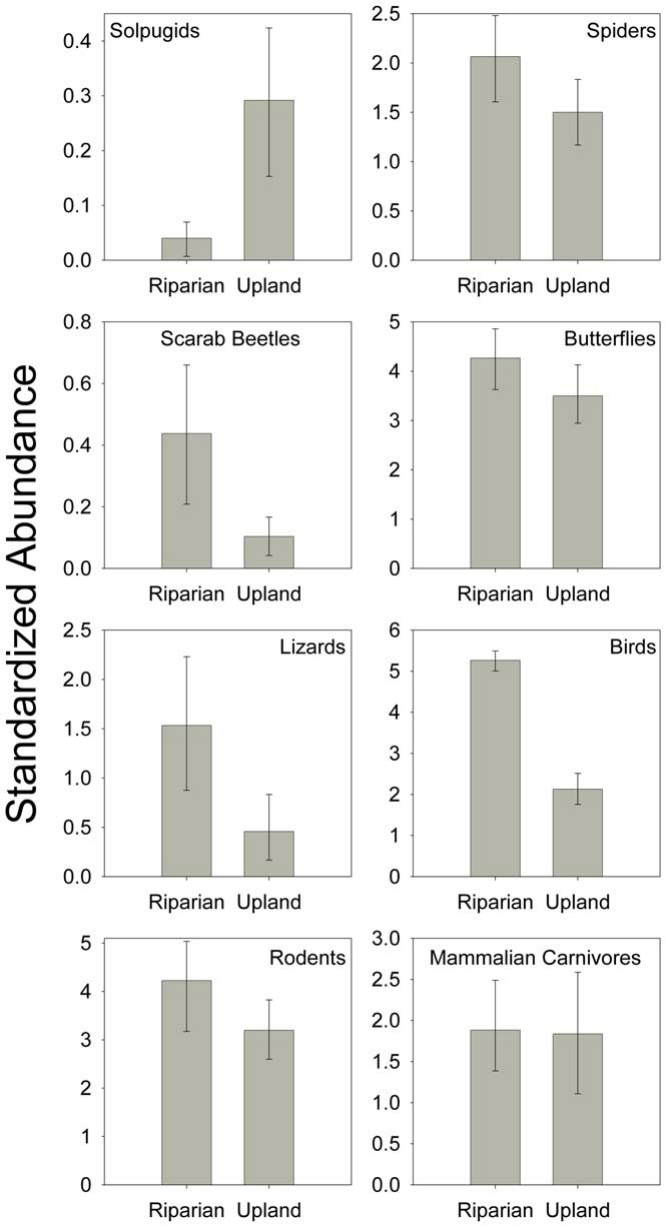
Abundance by zone for each taxonomic group. Bootstrapped abundance estimates (±95% confidence intervals) for the eight taxa sampled in both riparian and upland zones along the upper San Pedro River.

Three taxonomic groups (scarab beetles, lizards, and birds) were significantly more abundant in riparian than upland zones, while solpugids showed the opposite pattern (Figure 6). An additional four groups (spiders, butterflies, rodents, and mammalian carnivores) were more abundant in riparian zones, but the differences were not statistically significant.

Sørensen similarity between riparian and upland zones varied among taxa, ranging from as low as 0.166 for scarab beetles up to a maximum value of 1.0 for mammalian carnivores (Table 2). Half of the values were below 0.5 indicating overall low similarity among zones.

### Cross-Taxa Analyses

**Figure 7:**
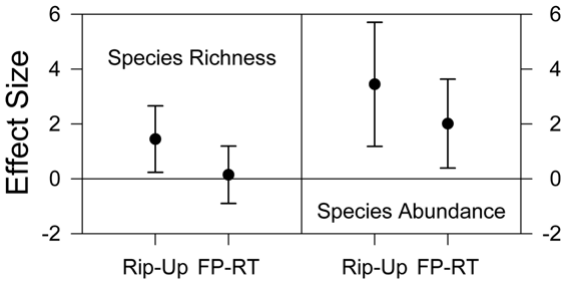
Richness and abundance meta-analysis results. Rip-Up = the difference in richness or abundance between riparian and upland zones; FP-RT = the difference in richness or abundance between river floodplain and river terrace habitat types. Results from the meta-analyses examining the difference in species richness and species abundance between riparian and upland zones (n = 8 groups) and between river floodplain and river terrace habitat types (n = 11 groups). The figure shows mean weighted effect sizes with associated upper and lower 95% confidence intervals.

Species richness was significantly greater in riparian versus upland zones (mean weighted effect size = 1.4471, n = 8, p = 0.0193; Figure 7), but not in river floodplain versus river terrace habitat types (mean weighted effect size = 0.1456, n = 11, p = 0.7846; Figure 7). Species abundance was significantly greater in riparian versus upland zones (mean weighted effect size = 3.4447, n = 8, p = 0.0028; Figure 7), and in river floodplain versus river terrace habitat types (mean weighted effect size = 2.0112, n = 11, p = 0.0149; Figure 7).

Mean similarity between habitat type pairs and zones (Table 2) ranged from a high of 0.622 (NS-FS) to a low of 0.310 (FP-FS), and generally decreased with increasing distance between habitat type pairs. However, relatively large variance estimates meant that the 95% confidence intervals for all similarity estimates overlapped.

### Trait-Level Analyses

Only one comparison was statistically significant at an alpha level of 0.05, abundances of taxa with different lifespans in FP vs. RT habitat types (Table 4). In other words, most grouping of taxa according to biological traits (e.g., by size or trophic level) did not differ significantly in richness or abundance between zones or between FP and RT habitat types. The relationships between biological traits and abundance/diversity patterns can be seen in the Supporting Information (Figures S1, S2, S3, S4, S5).

**Table 4:** Kruskal-Wallis test results (p-values) for between-habitat type or between-zone effect size differences based on the biological groups described in the Supporting Information: Table S1.

	Species Richness		Species Abundance	
Trait	Rip-Up	FP-RT	Rip-Up	FP-RT
Trophic Level	0.200	0.845	0.895	0.633
Body Size	0.853	0.365	0.363	0.190
Life Span	0.141	0.158	0.813	0.040
Thermoregulatory Regime	0.689	0.101	0.895	0.334
Taxonomic Affiliation	0.797	0.451	0.604	0.294

## Discussion

Understanding patterns of biodiversity across landscapes is one of the central pursuits of community ecology and relevant to a more effective theory of conservation. Here we show that riparian zones and near-river habitat types not only harbor more individuals and species than uplands, but also harbor different species altogether, elevating richness of the regional species pool. This observation provides a compelling middle ground between the conventional wisdom (riparian zones harbor more species) and a recent meta-analysis that suggested that riparian zones harbor different not more species. Specifically, our data suggest that previous empirical studies of riparian–upland richness gradients produced variable results (higher, equal and lower richness in riparia) due to a more narrow focus on single taxonomic groups. Our broader survey of the community along the San Pedro River suggests that in spite of variability at the level of individual taxonomic groups, the community as a whole demonstrated not only increased richness and abundance in riparian zones, but also high turnover between riparian zones and nearby upland ecosystems.

These results are particularly noteworthy given that many of the studies had their “desert” plots very near the riparian zone (only four taxa were sampled in desert scrub far from the river) which could mean that increased abundance and diversity often observed near edges might have eroded the measurable differences between the riparian and desert zones [31]. These edge effects could have manifested themselves in one of two ways: 1) species accumulation at the boundary of two habitat types, each of which provides critical resources (i.e., food and water); or 2) mass effects—also referred to as spillover—defined as the movement of individuals from favorable areas, or sources, to unfavorable areas, or sinks [32], [33]. The former mechanism would elevate species richness at habitat boundaries, such as the near scrub or river terrace habitat types that form the boundary between riparian and upland zones. The latter mechanism would be expected to elevate species richness in habitat types that share a border with multiple other habitat types, provided that each habitat type has a distinct assemblage of species.

In spite of these homogenizing forces, the low estimates of community similarity between riparian and upland zones provide support for the hypothesis that riparian zones contain a distinct suite of species that differ from those that occur in the surrounding uplands [11]. This result is especially noteworthy given the narrow width of riparian zones and how little area they occupy at a landscape scale. A still unresolved question involves whether riparian species in this semi-arid environment are evolutionarily distinct, or taxa that also occupy upland zones in more mesic regions. This question could potentially be addressed using museum records that are increasingly digitized and available in large repositories [34].

Though we omitted temporal variation in diversity patterns from this analysis, Stromberg [35] showed that the relative richness of plant communities in different habitat types along the lower San Pedro River changes seasonally in response to abiotic factors (flooding and precipitation) that affect the distribution and abundance of annual plants. Although not presented herein, between-year Sørensen similarity levels for butterflies and forbs/grasses were comparable to between-habitat type values for those two groups, suggesting that temporal turnover is important, particularly for short-lived, speciose groups such as insects and annual plants. Future work should strive to more explicitly integrate spatial and temporal patterns of species diversity.

The eleven groups surveyed in this study illustrate the variability in riparian–upland diversity patterns across taxa, with some groups being more species rich in the uplands, whereas others were more speciose in riparian zones (though only one of the contrasts approached statistical significance). The patterns are even more variable at the level of habitat type (as defined in the methods), with different taxa peaking in richness in river floodplain, river terrace, and near-river desert scrub habitat types. Likewise, community similarity between zones ranged from 0.166 to 1 (on a scale from 0 to 1) indicating considerable variability in species turnover between zones for different taxonomic groups.

With one exception, the heterogeneity in responses among taxa could not be attributed to the biological traits considered in this study. It may be that the biological traits we considered are not relevant to species richness and abundance patterns along riparian–upland gradients. However, we suspect one or more of the following factors affected the trait-level analysis. Firstly, it could be that our results are due to small sample sizes (8 or 11 taxa, depending on the comparison), making it difficult for any patterns that exist to emerge. It is also possible, that we looked for patterns at the wrong level and should have considered differences in traits within taxonomic groups rather than across taxa. Finally, it may be that the processes structuring communities along the riparian–upland gradient operate on suites of traits rather than individual traits in isolation. Unfortunately, the limited number of taxa included in this study precludes multivariate analyses that might identify relevant sets of traits.

The six factors described earlier—environmental stress, disturbance, competition, predation, biological productivity, and habitat structural complexity—can be invoked independently, or in concert to explain patterns in community structure along the riparian–upland gradient. Differences in the relative importance of these factors for each taxonomic group (or even individual species) may explain the variability in diversity patterns along the gradient. For example, certain taxa (i.e., those with limited mobility like plants) may be more sensitive to disturbance; other taxa may be insensitive to predation due to their position at the top of the food web (e.g., mammalian carnivores). The combined effect of these factors on species diversity along the riparian–upland gradient is hard to predict, though certain theories can be invoked (e.g., the dynamic equilibrium hypothesis [21], [36]), and tend to make predictions consistent with the results of this analysis (greater species richness in riparia with high turnover between zones).

Rigorously testing the effects of each factor on species diversity and/or the predictions of mechanistic theories is beyond the scope of this synthesis. However, we consider mechanistic studies in riparian landscapes to be an important avenue for future ecological research. The study of ecological processes at regional and geographic scales is complicated by logistic constraints and the need to make simplifying assumptions. In contrast, the species richness and community similarity patterns demonstrated in our study suggest that processes thought to structure ecological communities at large spatial scales also act at a meso-scale along riparian–upland gradients. Thus, riparian zones provide a compelling natural laboratory for testing some basic tenets of ecological theory [37].

### Conclusions

Our results indicate that species richness and abundance patterns along riparian–upland gradients vary among taxa (even within the same study system). This variability provides at least a partial explanation for the contrasting results of previous studies. However, when considered across taxa, species richness and abundance of the community as a whole is greater in riparian versus upland zones in this system. Furthermore, species turnover between riparian and upland zones is high, further elevating regional species richness in areas with intact riparian habitat.

Given the growth in human population along the San Pedro, and the concomitant demands for water, our results underscore the importance of conserving riparian areas and the hydrological processes that sustain them. Our results further highlight the unique flora and fauna that occupy upland zones, drawing attention to the need for conservation along the entire riparian–upland gradient. Without an intact gradient, the elevated biodiversity of the San Pedro watershed (and others like it) would be significantly diminished.

## Supporting Information

Detailed Methods S1 Taxon-specific survey methods used to gather information on species diversity in the different habitat types.
(DOC)

Figure S1 **Trophic level-based groupings for the riparian–upland and floodplain–river terrace (FP-RT) comparisons.** FP = river floodplain habitat type; RT = river terrace habitat type. Each point represents the unweighted effect size for a single taxonomic group belonging to one of the groupings. None of the trophic level comparisons were statistically significant at an alpha level of 0.05.
(TIF)

Figure S2 **Body size-based groupings for the riparian–upland and floodplain–river terrace (FP-RT) comparisons.** FP = river floodplain habitat type; RT = river terrace habitat type. Small ∼0.1–10 grams; Medium ∼10–1000 g; Large ∼1–100 kg. Each point represents the unweighted effect size for a single taxonomic group belonging to one of the groupings. None of the body size comparisons were statistically significant at an alpha level of 0.05.
(TIF)

Figure S3 **Lifespan-based groupings for the riparian–upland and floodplain–river terrace (FP-RT) comparisons.** FP = river floodplain habitat type; RT = river terrace habitat type. <1 yr = less than one year; ∼1 yr = approximately one year; >1 yr = greater than one year. Each point represents the unweighted effect size for a single taxonomic group belonging to one of the groupings. There was a statistically significant difference in abundance between river floodplain and river terrace habitat types for lifespan-based groups (at an alpha level of 0.05).
(TIF)

Figure S4 **Thermoregulatory mechanism-based groupings for the riparian–upland and floodplain–river terrace (FP-RT) comparisons.** FP = river floodplain habitat type; RT = river terrace habitat type. Each point represents the unweighted effect size for a single taxonomic group belonging to one of the groupings. None of the thermoregulatory mechanism comparisons were statistically significant at an alpha level of 0.05.
(TIF)

Figure S5 **Taxonomy-based groupings for the riparian–upland and floodplain–river terrace (FP-RT) comparisons.** FP = river floodplain habitat type; RT = river terrace habitat type. Each point represents the unweighted effect size for a single taxonomic group belonging to one of the groupings. None of the comparisons were statistically significant at an alpha level of 0.05.
(TIF)

Table S1 Biological groupings for the trait-level analyses.
(DOC)

Table S2 Number of surveys/samples and number of individuals detected by habitat type.
(DOC)
