# Supplementary material for: Multitaxonomic Diversity Patterns along a Desert Riparian–Upland Gradient
Source: PLoS One. 2012 Jan 17;7(1):e28235. doi: 10.1371/journal.pone.0028235 (PMC3260129; doi:10.1371/journal.pone.0028235)
Supplement: Detailed Methods S1 — Taxon-specific survey methods used to gather information on species diversity in the different habitat types. (DOC) [file pone.0028235.s001.doc]

*Forbs/Grasses, Shrubs, and Trees*

At each site, we established three, 20m wide belt transects spaced 100m apart. The transects were perpendicular to the river drainage, and each spanned the width of the river floodplain and extended for 100 meters onto the river terrace (pre-entrenchment alluvium) on either side of the river. Along each transect, we delineated discrete patches based on observed vegetative discontinuities in floristic structure (dominant woody species) and physiognomy (tree stem size, and percent cover within the canopy [>5 m], mid-story [1-5 m] and ground [<1 m] layers). Length of each patch along the transect line was measured. Herbaceous vegetation was sampled in August of 2001 and 2002, along two transect lines per site. Data were collected in August (the summer wet season) because it is time of peak seasonal biomass. Herbaceous cover, by species, was sampled in 1-m^2^ plots distributed along the transect lines. Plots were located along the transect lines in stratified random fashion (one plot per patch). If a patch was longer than 25 meters, an additional plot was sampled for each additional 25 meters of the patch. Woody vegetation was sampled along three transect lines. A 10x20m quadrat (long axis perpendicular to the transect) per patch was sampled for tree stem density and basal area, by species. Shrub cover was sampled along two line intercepts nested within the quadrat.

*Solpugids, Spiders, and Scarab Beetles*

These taxa were collected in pitfall traps set between May 2004 and October 2005. However, only spiders from May – October 2005 were identified to species and included in this study. A total of 64 pitfall traps were set at monthly intervals for two consecutive nights, arranged spatially as follows. Two pitfall traps, separated by 5-25 meters, were placed inside a plot (within a given habitat type). Each habitat type at each site had two plots, separated by ~100 meters. Pitfall traps were set in a total of four habitat types at each site, river floodplain (FP), river terrace (RT), near-river upland areas (NS), and upland areas that were far from the river (FS). The habitat types at a given site were separated by anywhere from 25-2500 meters, depending on the habitat pair. Pitfall traps were set at a total of four sites, each separated by about 10 km, for a total of 2 pitfalls per plot x 2 plots per habitat type x 4 habitat types per site x 4 sites per month = 64 pitfalls per month. Individual pitfall traps consisted of shallow plastic cups (about 10 cm deep and 7.5 cm across) filled about ¾ full with soapy water and buried flush with the ground surface. The cups were drained upon collection, and the samples were put in whirlzip bags filled with 70% ethanol for future identification.

*Butterflies*

We surveyed 9 study areas during a four-year period (1998-2001) throughout the 70 km extent of the SPRNCA with one additional site north of the boundary. Not all sites were sampled in all years due to wildfire. Most study areas were at least 1 km from the next closest area. For purposes of analysis, each study area was considered to be one independent sample site within the boundaries of the SPRNCA. Transects, placed perpendicular to edges between habitat types, consisted of contiguous 10 x 10 m plots that started at the habitat boundary and extended into habitat on either side of the edge. Only plots between 20 – 50m from the edge were used for these analyses in order to reduce edge effects.

Butterfly surveys were conducted from mid-August through early October in 1998 - 2001. From two to four complete rounds of surveys were conducted during each year with a survey round lasting three to four weeks. Surveys began at one end of the transect, with an observer surveying alternate plots to the end of the transect, then reversing direction and surveying the remaining plots on the way back. The starting point of the transect was alternated between rounds. Surveys were only begun when the sun was not obscured by clouds. Each 10 x 10 m plot was searched for 3 minutes and all butterflies seen were identified and recorded.

*Lizards*

We used three different approaches to surveying lizards: transects, plots, and large pitfall traps. We collected transect data during the summer of 2004 using distance sampling on line transects. We selected 3 sites along the river and placed a 100m transect in each of the four habitat types along the gradient: FP, RT, NS, and FS. Each transect was sampled using binoculars and a measuring tape, and each site was sampled four times from June – October 2004. Plot sampling was done in May of 2002. Three replicate 10 by 25 meter plots were set out in each of four habitat types (Cobble Bars, FP, RT, and NS), and monitored four times during the month. Pitfall sampling was done in June of 2002. Five-gallon buckets were buried flush with the ground in the center of each plot described below. Three, 5m-long arms of aluminum flashing were buried in the ground and positioned so as to guide lizards that encountered them towards the open pitfall buckets. The pitfalls were monitored multiple times each day to make sure that captured animals did not die from overheating.

*Birds*

Point-count data were collected at 74 sampling locations on 15 sites in the upper reaches of the San Pedro River. Each site consisted of point count locations located perpendicular from the river extending through river floodplain and river terrace habitat types and into the surrounding desert scrub. Points were located ≥100 m apart from one another and >60 m from a given edge type within the riparian corridor so that species richness estimates reflect community attributes pertaining to a given habitat type.

Each point represented the center of a variable circular plot extending to 50m radius to minimize the influence of adjacent habitat types in the species richness estimation. Observers recorded all birds seen or heard during an average of 8 visits per point during the 1998–2001 field seasons from May 15 to July 31. A total of 9 experienced observers conducted surveys during the 4 year study. At the beginning of each field season, a minimum of 2.5 weeks of training on survey techniques, including identification of birds by sight and sound, was done prior to data collection. Within a given year, observers were rotated between sites so that each point count location was surveyed approximately the same number of times by each observer.

*Small Mammals*

We used two different approaches to trapping small mammals: transects and quadrats. From July to August of 2003 we trapped small mammals across a range of sites and habitat types using the transect method. For this spatially extensive small mammal trapping we selected three study sites, each separated by >5 km. At each site we selected two sets of locations, separated by >500 meters for trapping. We set out four transects at each location, one in each of the following habitat types: FP, RT, NS, and FS, recording the spatial location of each transect using a hand-held GPS unit. Using Sherman live traps baited with a mixture of peanut butter and oats, we set the traps before sunset and processed all captures early the next morning. We set 40 traps per transect and trapped each location once in each of three time periods in early July, late July, and early August for a total of 2880 trap-nights.

In 2005 we trapped at four new sites, each separated by >5 km. As in 2003 we trapped in four different habitat types at each of the sites (using the same plots described above in the solpugids, spiders, and scarab beetles section). We trapped two plots per habitat type per site for a total of 32 plots. In each plot we baited and set ten Sherman live traps; we trapped during both the dry (June) and the post-monsoon season (October) for a total of 640 trap-nights in 2005.

*Mammalian Carnivores*

Ten transects were chosen within the SPRNCA that encompassed most of the river from the Mexico border to Willow wash.  Most transects consisted of loops that included a road or trail in the scrub/grassland next to the river and a return route within the riparian gallery of the riverbed.  Routes ranged from 1.9 to 11.8 km long.  Each transect was survey at least 3 times between October 1998 and January 2000; four transects were surveyed 4 times.  At least 2 months separated surveys on any individual route.  Surveys were conducted at least 2 weeks after any precipitation.  Tracks were identified by comparison with published sources (Murie 1975, Aranda 1981, Resendez, 1992, Orloff et al. 1993) and foot measurements from live-trapped and road killed animals.  Each time sign was encountered, it was identified to genus (hooded or striped skunks) or species (everything else).  Location was determined using a GPS receiver in conjunction with a USGS 7.5 minute topographic map. Habitat type was assigned to each sign using a GIS layer based on vegetation type. During each survey, data were recorded for the sign location only if it was > 1 km from previously recorded sign for that species, or it was in a different habitat type. This was done to protect against the chance of recording sign from the same individual twice for a given transect.

**References**

Aranda Sanchez, J.M. 1981. Rastros de los mamiferos silvestres de Mexico. –– Instituto National

de Investigaciones sobre Recursos Bioticos.

Murie, O.J. 1975. A field guide to mammal tracks, 2^nd^ edition. –– Houghton Mifflin Company.

Orloff, S.G., Flannery, W. and Belt, K.C. 1993. Identification of the San Joaquin kit fox (*Vulpes*

*macrotis mutica*) tracks on aluminum tracking plates. –– California Fish and Game 79: 45-53.

Resendez, P. 1992. Tracking and the art of seeing. How to read animal tracks and sign. ––

Camden House Publishing, Inc.
