# Supplementary material for: Multitaxonomic Diversity Patterns along a Desert Riparian–Upland Gradient
Source: PLoS One. 2012 Jan 17;7(1):e28235. doi: 10.1371/journal.pone.0028235 (PMC3260129; doi:10.1371/journal.pone.0028235)
Supplement: Table S1 — Biological groupings for the trait-level analyses. (DOC) [file pone.0028235.s007.doc]

**Table S1.** Biological groupings for the trait-level analyses.

| **Trait** | **Group 1** | **Group 2** | **Group 3** |
| --- | --- | --- | --- |
|  |  |  |  |
| Trophic level | *Primary Producers* | *Herbivores* | *Carnivores* |
|  | Forbs & Grasses | Butterflies | Solpugids |
|  | Shrubs | Herbivorous Birds | Spiders |
|  | Trees | Rodents (w/o *Onychomys*) | Lizards |
|  |  |  | Carnivorous Birds |
|  |  |  | Mammalian Carnivores |
|  |  |  |  |
| Body Size | *~0.1 - 10 grams* | *~10 - 1000 g* | *~1 - 100 kg* |
|  | Solpugids | Forbs & Grasses | Shrubs |
|  | Spiders | Lizards | Trees |
|  | Scarabs | Birds | Mammalian carnivores |
|  | Butterflies | Rodents |  |
|  |  |  |  |
| Life Span | *< 1 year* | *~ 1 year* | *> 1 year* |
|  | Annual Forbs & Grasses | Solpugids | Perennial Forbs & Grasses |
|  | Spiders | Lizards | Shrubs |
|  | Butterflies | Rodents | Trees |
|  | Scarabs |  | Birds |
|  |  |  | Mammalian Carnivores |
|  |  |  |  |
| Thermoregulatory Mechanism | *Ecothermic* | *Endothermic* |  |
|  | Solpugids | Birds |  |
|  | Spiders | Rodents |  |
|  | Scarabs | Mammalian Carnivores |  |
|  | Butterflies |  |  |
|  | Lizards |  |  |
|  |  |  |  |
| Taxonomic Affiliation | *Plants* | *Invertebrates* | *Vertebrates* |
|  | Forbs & Grasses | Solpugids | Lizards |
|  | Shrubs | Spiders | Birds |
|  | Trees | Scarabs | Rodents |
|  |  | Butterflies | Mammalian Carnivores |
