# Supplementary material for: Multitaxonomic Diversity Patterns along a Desert Riparian–Upland Gradient
Source: PLoS One. 2012 Jan 17;7(1):e28235. doi: 10.1371/journal.pone.0028235 (PMC3260129; doi:10.1371/journal.pone.0028235)
Supplement: Table S2 — Number of surveys/samples and number of individuals detected by habitat type. (DOC) [file pone.0028235.s008.doc]

**Table S2.** Number of surveys/samples and number of individuals detected by habitat type.

|  | **Number of Samples/Surveys** | | | | **Number of Individuals*** | | | | **-------------------- Number of Species --------------------** | | | | | | |
| --- | --- | --- | --- | --- | --- | --- | --- | --- | --- | --- | --- | --- | --- | --- | --- |
| **Taxonomic Group** | **FP** | **RT** | **NS** | **FS** | **FP** | **RT** | **NS** | **FS** | **FP** | **RT** | **NS** | **FS** | **Riparian** | **Upland** | **Total** |
| Forbs & Grasses | 365 | 255 | NA | NA | 1705 | 1068 | NA | NA | 134 | 117 | NA | NA | 161 | NA | 161 |
| Shrubs | 235 | 134 | NA | NA | 238 | 73 | NA | NA | 17 | 17 | NA | NA | 21 | NA | 21 |
| Trees | 235 | 134 | NA | NA | 312 | 105 | NA | NA | 10 | 4 | NA | NA | 11 | NA | 11 |
| Solpugids | 72 | 72 | 72 | 72 | 1 | 5 | 25 | 18 | 1 | 5 | 6 | 4 | 5 | 6 | 8 |
| Spiders | 24 | 24 | 24 | 24 | 52 | 48 | 28 | 45 | 23 | 26 | 15 | 22 | 43 | 32 | 59 |
| Scarab Beetles | 72 | 72 | 72 | 72 | 41 | 18 | 5 | 10 | 11 | 8 | 4 | 6 | 16 | 12 | 22 |
| Butterflies | 142 | 181 | 162 | NA | 515 | 857 | 565 | NA | 31 | 40 | 28 | NA | 44 | 28 | 44 |
| Lizards | 24 | 24 | 24 | NA | 43 | 31 | 11 | NA | 4 | 6 | 6 | NA | 8 | 6 | 11 |
| Birds | 278 | 229 | 82 | NA | 1587 | 1075 | 152 | NA | 58 | 47 | 29 | NA | 66 | 29 | 74 |
| Rodents | 26 | 26 | 26 | 26 | 110 | 106 | 80 | 87 | 7 | 8 | 7 | 6 | 8 | 7 | 9 |
| Mammalian Carnivores | 32 | 35 | 38 | | 101 | 141 | 184 | | 12 | 10 | 12 | | 12 | 12 | 12 |

Note: FP = river floodplain habitat types; RT = river terrace habitat types; NS = upland areas within 1km of the river; FS = upland areas greater than 1km from the river. The mammalian carnivore data were collected without categorizing the upland areas as near- or far-from the river.

* Plant data were collected as percent abundance. Therefore, the minimum number of individuals was estimated by assigning all cover for a given species in a given plot to the same individual.
